# A Positive Impact of Sport-Related Musculoskeletal Pain on the Quality of Life and Pain Attitudes in Adolescents with Lower-Limb Disability

**DOI:** 10.3390/healthcare13222856

**Published:** 2025-11-10

**Authors:** Anna Gogola, Maciej Biały, Rafał Gnat

**Affiliations:** Institute of Physiotherapy and Health Sciences, Academy of Physical Education, 40-065 Katowice, Poland; aniagogola@op.pl (A.G.); mbfizjoterapia@gmail.com (M.B.)

**Keywords:** sport, para-sport, quality of life, pain, pain attitude

## Abstract

**Background/Objectives:** Sport participation provides important physical and psychological benefits but carries a risk of musculoskeletal injury and associated pain. In adolescents with lower-limb disabilities, sport-related pain may exert a distinct influence on quality of life, coping strategies, and ongoing engagement in physical activity. This study examines these effects in sitwake training participants, comparing adolescents who experienced transient injuries during training with those who did not, and evaluates subsequent changes in pain attitudes, quality of life, and return-to-sport rates. **Method:** A prospective case–control study was conducted over three months, with baseline and follow-up assessments. Adolescents with lower-limb disabilities engaged in sitwake training were assigned to a pain group (PG: with transient injuries with moderate pain during the observation period) or a no-pain group (no-PG). Health-related quality of life and pain attitudes were measured using the 37-item DISABKIDS Chronic Generic Module and the 27-item Pain Attitudes Questionnaire. Injuries in PG were managed with physiotherapy. Return-to-training rates were recorded the following season. **Results**: At baseline, the PG and no-PG showed comparable scores in the two scales. By follow-up, the PG demonstrated significantly greater improvements in independence, physical limitations, emotions, social inclusion, stoicism, and cautiousness subscales (all inter-group change score differences: *p* ≤ 0.001). No differences were observed for social exclusion (*p* = 0.229) or treatment impact (*p* = 0.986) subscales. Return to training rates were higher in the PG (65%) than no-PG (29%). **Conclusions**: Sitwake training participation with transient, moderate pain promotes positive psychosocial outcomes in adolescents with lower-limb disabilities. The PG showed greater improvements in quality of life and adaptive pain attitudes, with higher return-to-training rates. Structured, manageable sport-related pain may facilitate resilience, coping, and sustained engagement in sport.

## 1. Introduction

Sport and physical activity are fundamental to maintaining health across all age groups. Their benefits are extensive, including chronic disease prevention, reduced morbidity and mortality, and improved mental well-being [1,2]. However, participation also carries a risk of injury. Sports injuries most commonly involve the musculoskeletal system and typically present as tissue damage and/or pain [3,4]. Common examples, including muscle strains, ligament sprains, and overuse injuries, frequently cause acute pain, limit movement, reduce force generation, and impair coordination, thereby compromising overall motor performance [5,6]. These symptoms are consistently observed across a wide range of sports and levels of competition, from recreational to elite [6,7,8].

Injuries represent a complex challenge that extends beyond biological processes alone. Athletes, particularly those engaged in contact-, strength-, or endurance-based disciplines, may develop an elevated tolerance to pain through repeated exposure; however, they remain susceptible to significant psychological consequences [9,10]. A large body of research highlights the adverse psychological sequelae of injury. Empirical evidence indicates that injuries are frequently associated with heightened stress, anxiety, depressive symptoms, and reduced self-esteem, all of which may compromise motivation, adherence to rehabilitation protocols, and return-to-play outcomes [5,11,12]. Moreover, retirement from sport—particularly when precipitated by injury or deselection—can have profound and sustained effects on mental health [13]. Thus, while athletes may appear physically resilient, the psychological repercussions of injury represent a considerable challenge, underscoring the need for holistic management approaches that address both physical and psychological dimensions [14,15,16,17,18].

The causes, consequences, and management of sports injuries have predominantly been investigated in able-bodied athletes, with research on athletes with physical disabilities remaining comparatively limited. In para-sport, however, pain and its implications acquire a distinct and multifaceted dimension [19,20]. Pain perception in disabled athletes is influenced not only by sporting exposure, but also by factors such as spasticity, postural deviation, and reliance on assistive devices [19]. Repeated exposure to discomfort in daily life—for example, arising from spasticity or altered posture—may further contribute to modified pain experience. Although the physiological aspects of pain responses broadly resemble those of able-bodied athletes, reports more frequently highlight a beneficial psychological dimension of sport-related pain in disabled sportsmen. Evidence suggests that pain may contribute to emotional resilience and psychological adaptation through complex, multifactorial processes. In competitive athletes with spinal cord injury, resilience appears to be supported by pre-existing personality traits, social support, coping strategies, and motivation to adapt, with sport participation itself facilitating adjustment to acquired disability [21]. Behavioural patterns may also diverge; disabled athletes have been shown to report lower levels of antisocial behaviour and moral disengagement compared with their able-bodied counterparts [22,23]. Within this context, pain may assume a constructive role. While intrinsically aversive, it has the potential to support positive changes in self-perception and self-esteem, thereby enhancing quality of life, emotional growth, and motivation to maintain physical activity. Sport- and competition-related pain—distinct from that associated with illness—may also elicit a more challenge-oriented emotionality, whereby discomfort is interpreted as a stimulus for perseverance and personal growth rather than a source of distress. This may be particularly salient among individuals vulnerable to negative body perceptions arising from health conditions, aesthetic concerns, and disability.

Despite growing recognition of these adaptive mechanisms in adult para-athletes, far less is known about adolescents with disabilities. This population may not yet have fully developed strategies for managing pain, rendering them particularly vulnerable to maladaptive responses such as depressive symptoms, social withdrawal, or disengagement from physical activity [2,6,24]. Simultaneously, adolescence represents a critical developmental stage in which sport participation—including exposure to mild to moderate sport-related pain—may operate as a form of ‘adaptive training’, shaping more constructive attitudes towards discomfort and fostering resilience. It is important to emphasise that such pain should occur only within controlled, supervised, and therapeutically guided contexts, where its intensity and duration remain manageable and recovery is appropriately monitored. Within these boundaries, experiencing and overcoming transient injury and pain may help prevent the emergence of negative behavioural and emotional patterns, including depressive or antisocial tendencies, which are reported more frequently among youth with disabilities than among their able-bodied peers.

The present study addresses this research gap by examining adolescents with lower-limb disabilities who began participating in a newly emerging para-sport discipline—adaptive wakeboarding, also known as sitwake (see Section 2.4). The aims of this study, which are all of equal priority, are to (1) evaluate changes in quality of life among adolescents with lower-limb disabilities participating in sitwake training, comparing those who experienced transient, training-related injuries associated with moderate pain with those who did not; (2) examine changes in pain attitudes in these two groups; and (3) determine return-to-sitwake training rates in the following season. We hypothesised that the occurrence of injuries, despite the accompanying pain, would be associated with beneficial changes in quality of life and pain attitudes, thereby increasing the likelihood of continued participation.

## 2. Materials and Methods

### 2.1. Design and Participants

This was a prospective case–control study with a three-month observation period, during which the dependent variables were measured twice: at baseline, prior to observation, and again at the end of the study period. The study was approved by the Institutional Research Ethics Committee (No. 13/2020).

The minimum required sample size was determined by a Sample Size Calculator 2.0 [25] using formulas proposed by Naing [26], with the following assumptions: α = 0.05, minimum effect size = 15%. The largest requirement was calculated for the cautiousness subscale of the Pain Attitudes Questionnaire (n = 19) (see below). Recruitment was concluded once the minimum required sample size in the PG had been reached (n = 20).

Participants were recruited at a local wakeboarding club during the enrolment of young people with physical disabilities interested in sitwake training. Recruitment spanned three consecutive seasons (2021–2024), during which the procedures for recruitment, training, and therapeutic management of injuries remained identical. The programme targeted adolescents aged 12–18 years with physical disabilities.

Eligibility was verified during an introductory meeting and medical examination conducted by a physician, a physiotherapist, and a sitwake instructor. Inclusion criteria comprised age 12–18 years, first-time participation in sitwake training, lower limb disability precluding independent walking (supported upright posture only), full upper limb function and intellectual capacity, absence of comorbidities or psychological disorders limiting physical activity, and ≥70% adherence to the training programme.

Of 69 eligible individuals, 63 (with guardian consent) participated and completed baseline assessments. During the 3-month observation period (June-August in each season), participants who sustained sitwake-related musculoskeletal injuries with pain and functional impairment were classified into the pain group (PG). Inclusion criteria for this group were: mild to moderate injury, pain intensity of 50–80 mm on the Visual Analogue Scale (VAS; 0–100 mm), pain severe enough to suspend training for at least one week, and subsequent pain reduction to 0–10 mm on the VAS following therapy. Severe injuries requiring medical or orthopaedic intervention beyond physiotherapy or physical modalities (e.g., electrotherapy, ultrasound, cryotherapy) were excluded. Participants not meeting PG criteria were assigned to the no-pain group (no-PG).

**Figure 1:**
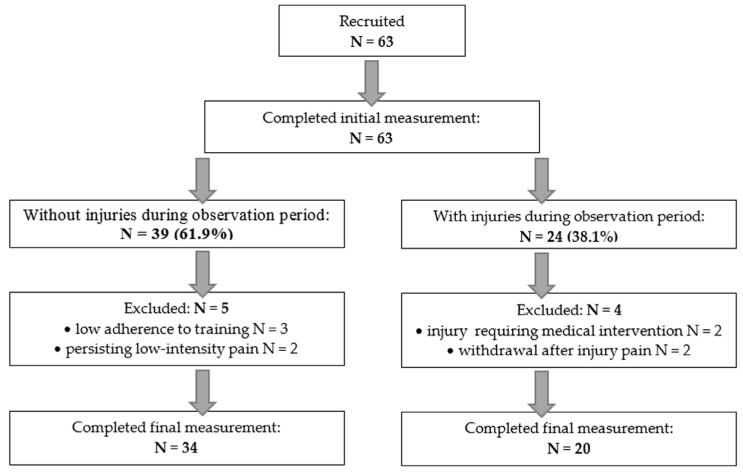
Participants flow through the consecutive stages of the procedure.

Across the three seasons, 24 participants sustained sitwake-related injuries. In total, 2 were excluded due to severity and 2 withdrew from training, yielding 20 participants in the PG, all of whom completed the final assessment. Among the 39 individuals initially assigned to the no-PG, 2 were excluded for transient post-exercise pain (VAS 30–40 mm) and 3 for adherence below 70%, resulting in a final sample of 34 participants who completed all assessments. Participant flow is shown in Figure 1, and basic demographics are presented in Table 1.

Injuries in the PG primarily involved soft-tissue bruises, muscle strains, joint sprains, and non-sprain joint disorders (cartilage irritation, capsular stress), mainly affecting the shoulder girdle, upper arm, neck, and upper torso, typically following falls, transfers, or collisions. Occasional lower-limb injuries were also observed. Classification of injury severity followed the grade I/II definitions by Grassi et al. [27]. Grade I (mild) denotes injury with minimal functional loss, minimal swelling or pain, and full or near-normal range of motion, whereas Grade II (moderate) indicates partial structural or functional disruption, evident functional limitation, and reduced range of motion. The mean initial pain intensity was 63.45 mm (±7.74; 52–79) on the VAS scale, decreasing to 5.30 mm (±3.88; 0–10) at final assessment. The mean training absence due to injury was 12.35 days (±3.34; 8–19). The type and location of injuries recorded in the PG are detailed in Table 2.

### 2.2. Measurements

In order to perform an investigation, 2 standardised questionnaires were selected. They were the 37-item DISABKIDS Chronic Generic Module (DCGM-37) [28,29] and 27-item Pain Attitudes Questionnaire (PAQ-27) [30,31].

The DCGM-37 items are grouped into six subscales reflecting factors relevant to health-related quality of life in children and adolescents: independence, physical limitations, emotions, social exclusion, social inclusion, and treatment impact [32]. Items are rated on a five-point Likert scale reflecting the frequency of behaviours or feelings experienced over the preceding four weeks, with response options ranging from 1 (never) to 5 (always). For scoring, responses within each subscale are converted to a 0–100 scale, with 1 = 100, 2 = 75, 3 = 50, 4 = 25, and 5 = 0, and then summed. The DCGM-37 has demonstrated satisfactory internal consistency, with Cronbach’s alpha ranging from 0.70 to 0.87 across subscales, as well as good test–retest reliability, with intraclass correlation coefficients between 0.71 and 0.83 [28]. Higher scores indicate a greater influence of the respective factor on health-related quality of life. Greater influence in the independence and social inclusion subscales may be considered positive, whereas for physical limitations, emotions, social exclusion, and treatment impact, it is interpreted as negative.

For the PAQ-27, a two-factor model was used [33], comprising subscales of stoicism (14 items) and cautiousness (13 items). Within this framework, stoicism reflects behaviours such as reluctance to express pain and perceiving oneself as having superior control over pain, whereas cautiousness reflects uncertainty regarding whether a given sensation should be identified as pain and/or reluctance to label a sensation as painful. Each item was rated on a five-point Likert scale, ranging from 1 (strongly disagree) to 5 (strongly agree). The mean score of the items within each subscale was used as the outcome measure. In an Australian adult population, Yong et al. (2001) reported good to excellent internal consistency for the PAQ-27, with Cronbach’s alpha coefficients ranging from 0.78 to 0.86 for the stoicism subscale and from 0.75 to 0.81 for the cautiousness subscale [31]. Test–retest reliability was also demonstrated, with Pearson’s correlation coefficients ranging from 0.86 to 0.87 for stoicism and from 0.91 to 0.92 for cautiousness. Higher scores indicate a higher level of the respective pain attitude.

Return-to-sitwake training was recorded at the start of the season subsequent to the one in which the aforementioned measurements were obtained.

The DCGM-37 and PAQ-27 are established questionnaires that have been described previously [28,30,31,32]. Their Polish adaptations, including translation and linguistic validation, were conducted in accordance with the following protocol. Initially, two bilingual medical professionals, both native Polish speakers, independently translated the English versions into Polish. These translations were then reviewed by a panel of four specialists in paediatrics and psychology, who reconciled discrepancies and produced a single consensus version of each questionnaire. These drafts were subsequently subjected to back-translation by an independent bilingual medical expert, with the resulting texts re-evaluated by the panel. Following minor adjustments, a pilot study was conducted with 10 adolescents. After completing the questionnaires, participants were individually interviewed by one of the authors to verify comprehensibility, to identify any missing relevant content, and to assess the appropriateness of the response options.

### 2.3. Research Team

The authors assumed supervisory and coordinating roles, participating in participant recruitment, preparing online questionnaires, providing instructions during data collection, curating the databases, and conducting data analyses. They were not directly involved in training or therapeutic procedures.

The research team included two physiotherapists (Master of Science in rehabilitation, with additional training in physiotherapy, manual therapy, and massage) with 10–11 years of professional experience and approximately five years affiliated with a wakeboarding club. They assessed training candidates, monitored injuries and pain symptoms, and implemented therapeutic plans for participants assigned to the PG in consultation with a physician.

A medical doctor specialising in orthopaedics conducted initial screenings, examined PG participants, collaborated on therapeutic plans, and assessed readiness to return to training.

Two wakeboarding instructors, each with ten years of experience in sit-board sessions for individuals with disabilities, recruited participants and delivered all training sessions. Both are active athletes and leaders of the Paralympic Wake Camp.

All team members also participated in final debriefings at the end of each training season. The club manager and cable system operators supported logistics, technical, and organisational aspects of the training.

### 2.4. Sitwake Training

Sitwake, or sit-down wakeboarding, is an adaptive water sport tailored for individuals with lower-limb impairments. Athletes utilise a specially designed wakeboard equipped with a seated bucket and harness, providing stability while being towed by a cable system. The sport encompasses a wide range of on-water activities analogous to those in traditional wakeboarding, modified for the seated position. In the present study, participants acquired the fundamental skills required for sitwake, including controlled carving, turns, and directional changes, and progressed to basic acrobatic manoeuvres, such as simple jumps, spins, and grabs.

Over each of the three 13-week training seasons, a total of 39 sitwake training sessions were conducted. The sessions were delivered alternately by two instructors, depending on their individual availability. This arrangement did not cause major disruptions, as both possessed comparable skills and experience, and followed a standardised training programme. The proportion of sessions conducted by each instructor was similar, i.e., 46% and 54%.

Training sessions were held three times per week, each lasting 1.5 h. The structure comprised: (1) introduction (10–15 min): equipment checks and adjustment, preparatory warm-up including mobility drills, core activation, and balance exercises, supported by mental rehearsal and visualisation; (2) main component (60–75 min): on-water training aligned with the weekly thematic focus; (3) conclusion (10–15 min): structured stretching, relaxation, feedback, and systematic review of recorded performance. The training scheme remained uniform (Table 3) owing to the participants’ low skill level and the practical difficulty of forming subgroups of differing proficiency. Individual differences related to the relatively comparable degree of disability were addressed through small, ad hoc modifications to the planned exercises.

### 2.5. Therapy

Within the PG, therapeutic sessions with a physiotherapist were conducted every second day, excluding weekends, each lasting approximately 1.5 h. Sessions were delivered alternately by two physiotherapists, depending on availability. This arrangement did not cause disruption, as both possessed comparable expertise and adhered to the standardised PEACE & LOVE protocol [34,35], integrating acute and subacute treatment strategies. The distribution of sessions was slightly unbalanced, i.e., 39% and 61%. After each session, participants received specific recommendations for the intervening days and were required to follow these therapeutic instructions in the home setting.

All participants allocated to the PG initially reported pain intensity ≥50 mm on the VAS, at which point the PEACE phase was applied. Protection (P) involved limiting aggravating movements and, where required, using temporary supports such as slings or taping to allow tissues to settle. Short, pain-free movements were encouraged to prevent stiffness, as Protection did not equate to strict immobilisation. Elevation (E) was recommended where feasible, positioning the upper limb above heart level to promote venous return and minimise swelling. Avoidance of anti-inflammatory agents (A) was emphasised unless strictly necessary, with guidance to minimise their use. Compression (C) with elastic bandages was applied in cases of visible swelling, providing mechanical support and controlling oedema. Structured Education (E) was delivered during the first therapy session, covering injury mechanisms, expected recovery timelines, and the importance of active engagement in rehabilitation to promote adherence and self-efficacy.

Progression to the LOVE phase was initiated once pain intensity had decreased to approximately 20–30 mm on the VAS. Loading (L) was introduced through pain-free, individualised exercises, beginning with assisted movements and progressing to active strengthening. The optimism (O) component was deliberately omitted because this study aimed to measure variables of a psychological and emotion-related nature, involving subjective judgements concerning individual quality of life and pain attitudes. Including explicit optimism-focused therapeutic interventions could have influenced these outcomes, potentially confounding the effects of the sustained injuries and pain. By omitting this component, we sought to isolate the impact of sport-related pain while minimising therapist-induced bias. Vascularisation (V) was promoted through light aerobic activity adapted to the seated position (e.g., arm ergometry, rhythmic upper-limb movements) to enhance circulation and support metabolic repair. Exercise (E) focused on restoring joint mobility, proprioception, and sport-specific function, with progression to higher-intensity drills only once baseline control and confidence had been regained. By the end of the therapeutic process, pain reduction to 0–10 mm on the VAS was achieved.

Where clinically indicated, additional physical modalities were applied to support tissue healing, reduce pain, and enhance recovery. These included electrotherapy to reduce pain and improve circulation, magnetic field therapy to promote tissue repair, ultrasound for deep tissue heating, and cryotherapy to manage inflammation and alleviate pain.

### 2.6. Procedure

Prior to the first training session, all participants were asked to arrive one hour earlier to complete baseline measurements of the dependent variables. They first received detailed instructions regarding the DCGM-37, after which they read the questionnaire on a computer screen and entered their responses. To maintain adequate concentration, a 20 min break was scheduled, including a 10 min period of light rest, followed by instructions for the PAQ-27. Again, participants then completed the PAQ-27 on the computer. The entire procedure lasted approximately 40–50 min.

The next stage comprised a 13-week sitwake training period (see above). During this period, participants were instructed to report any occurrence of pain and/or dysfunction directly to the physiotherapists and to rate its intensity using the VAS. All participants with pain intensity exceeding 50 mm were immediately referred to the medical doctor for consultation. Those reporting pain intensity < 50 mm were monitored by physiotherapists, including follow-up by telephone. If pain intensity increased, the participant was referred to the medical doctor; if it decreased, continuation of training was recommended. For participants meeting the criteria for the PG, injuries were managed by the physiotherapy team as outlined above.

Within one week after the final training session, the dependent variables were reassessed during a summary visit at the wakeboarding club. During this visit, the entire team (authors, medical doctor, physiotherapists, and sitwake instructors) provided their concluding opinions, recommendations, and suggestions for the future to the participants and their legal guardians. The procedure of this follow-up data collection was identical to that employed during the initial assessment. A three-month interval was considered sufficient for participants to have forgotten their previous responses and to allow for a measurable change in the dependent variables [36].

### 2.7. Data Management

All responses were stored in a computerised database. The authors did not have access to the database until data collection was completed, in order to minimise potential bias. Scale scores were calculated strictly according to the original scoring instructions. Both baseline and follow-up scores, as well as change scores (calculated as follow-up minus baseline), were subjected to statistical analysis.

### 2.8. Statistical Analysis

To assess the internal consistency of the data, Cronbach’s alpha coefficient was used. Deviations from normal distribution were analysed using the Shapiro–Wilk test. Intra-group differences were assessed using the dependent data Student’s *t*-test or non-parametric Wilcoxon (dependent on data distribution), inter-group differences—using the independent data Student’s *t*-test or non-parametric Mann–Whitney test. Difference in return to training rates was evaluated using the statistical test for two fractions. The Statistica 13.0 software (StatSoft, Tulsa, OK, USA) was used.

## 3. Results

### 3.1. Internal Consistency

In the case of all the scales and subscales utilised, acceptable internal consistency was recorded (Cronbach’s alpha coefficients 0.70–0.90).

### 3.2. DISABKIDS Chronic Generic Module

**Figure 2:**
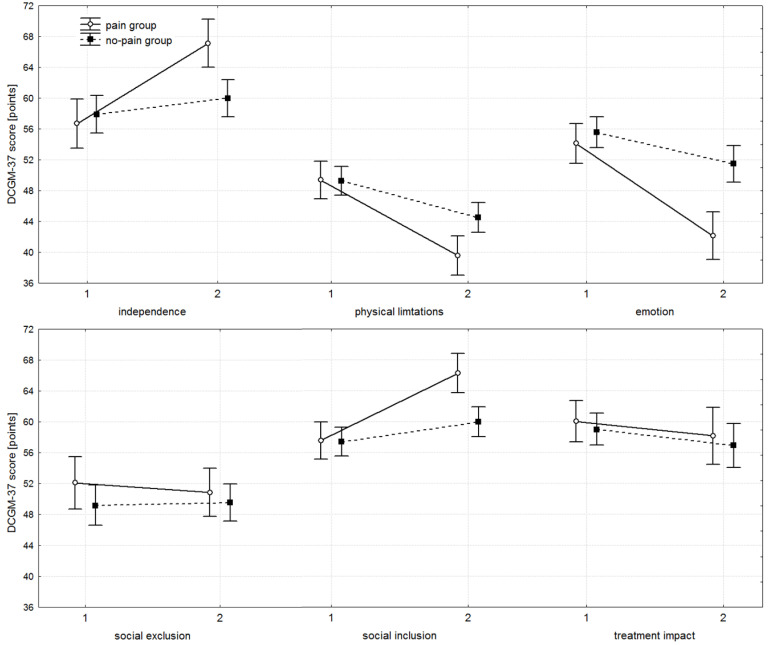
Mean scores with 95% confidence intervals (whiskers) for the subscales of the DISABKIDS Chronic Generic Module (DCGM-37) questionnaire, presented for the pain and no-pain groups. At baseline (1), no significant differences were observed between groups (all *p* > 0.05). At the follow-up measurement (2), significant differences emerged in the independence (*p* = 0.001), physical limitations (*p* = 0.003), emotion (*p* = 0.001), and social inclusion (*p* = 0.001) subscales, in each case favouring the pain group. No differences were found for social exclusion and treatment impact subscales (all *p* > 0.05).

For the independence, physical limitations, emotion, and social inclusion subscales of the DCGM-37, a similar pattern was observed. The PG and no-PG started from comparable baseline levels, with no significant inter-group differences (Table 4 and Figure 2). At follow-up, however, significant differences emerged in favour of the PG. Change scores for these subscales were likewise significantly greater in the PG. Both groups showed significant improvement between baseline and follow-up assessments. While both groups demonstrated statistically significant intra-group change, clinically meaningful improvement was observed almost exclusively in the PG. The PG yielded consistently large effect sizes (d > 1.0 in nearly all responsive domains, Table 5), large absolute change scores, and concordant statistically significant *p*-values. In contrast, the no-PG demonstrated only small effect sizes, indicating that statistically significant findings in this group were not accompanied by meaningful clinical benefit.

In contrast, for the social exclusion and treatment impact subscales, no significant inter- or intra-group differences were detected, either in absolute scores or in change scores.

### 3.3. Pain Attitudes Questionnaire

**Figure 3:**
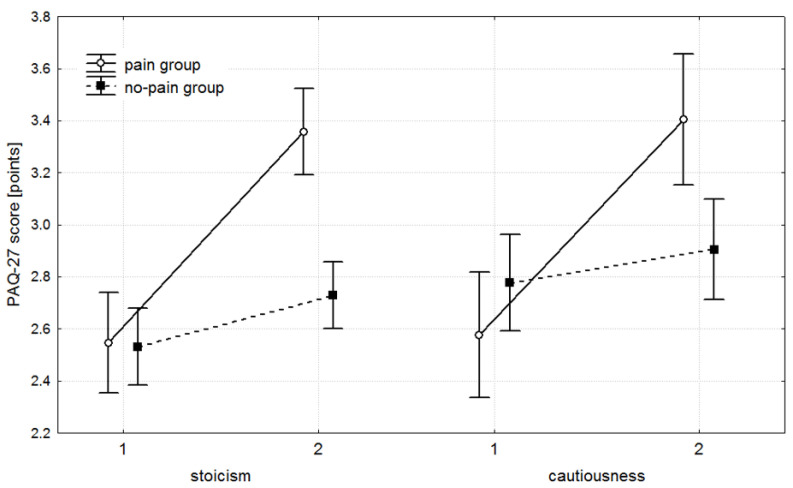
Mean scores with 95% confidence intervals (whiskers) for the subscales of the Pain Attitudes Questionnaire (PAQ-27) questionnaire, presented for the pain and no-pain groups. At baseline (1), no significant differences were observed between groups (all *p* > 0.05). At the follow-up measurement (2), significant differences emerged in the stoicism (*p* = 0.001) and cautiousness (*p* = 0.003) subscales, both in favour of the pain group.

For the stoicism and cautiousness subscales of the PAQ-27, a comparable trend was noted. At baseline, the PG and no-PG exhibited similar scores, with no significant differences between groups (Table 4 and Figure 3). By follow-up, significant inter-group differences had developed, again favouring the PG. The magnitude of change between baseline and follow-up was also significantly greater in the PG. Nonetheless, both groups demonstrated significant within-group improvements over time. Effect sizes for the two PAQ-27 subscales behaved as for the DCGM-37, with large effects in the PG and only small effects in the no-PG, reinforcing that clinically meaningful improvement was observed predominantly in the PG (Table 5).

### 3.4. Return to Training Rate

At the beginning of the season, following completion of the DCGM-37 and PAQ-27, 65% of participants in the PG (n = 13) and 29.4% in the no-PG (n = 10) continued sitwake training. The difference between these two fractions was statistically significant (test for two fractions; *p* = 0.011).

## 4. Discussion

The aim of this study was to examine whether transient injuries associated with moderate pain during sitwake training could influence adolescents with lower-limb disabilities in ways that go beyond the immediate biological function of pain. Specifically, we evaluated whether such experiences would be linked with changes in quality of life, shifts in pain attitudes, and subsequent likelihood of returning to the sport. In formulating this objective, we anticipated that pain—although inherently aversive—might under certain conditions serve as a stimulus for beneficial psychosocial adaptation and reinforce continued participation.

Our study deliberately does not dispute the fundamental biological role of pain as a tissue damage signal. Acute pain in the context of sport fulfilled this uncontroversial function in our sample by alerting participants to ongoing or recent tissue injury [37,38]. Rather, the novel and clinically relevant question addressed here concerns what follows the nociceptive signal—the psychosocial cascade that shapes interpretation, behaviour, and long-term participation.

Pain is not a unitary experience; it triggers highly variable psychological responses that are filtered through cognitive, emotional, and social processes [39]. Our findings align with literature showing that, beyond raw nociception, meaning-making, prior exposure, social support, self-efficacy, and coping repertoire largely determine whether pain becomes disabling or, conversely, an opportunity for growth [40,41]. In this sense, sport provides a specific context in which pain can be reappraised: when experienced in a framework of instrumental training and group belonging, transient sport-related pain may be interpreted as evidence of effort, progress, and resilience rather than solely as a catastrophic threat.

This mechanism helps explain our central observation—that moderate, transient training injuries were associated with favourable shifts in quality of life among adolescents with disabilities.

Interpreting the mean values recorded for both the DCGM-37 and the PAQ-27 is challenging because no established normative data exist for our specific population. Nevertheless, approximate interpretative anchors can be drawn from published field-testing and validation studies. Large-scale European field testing of the DCGM-37 in more than 1000 children and adolescents with chronic conditions reported subscale scores consistently above the metric midpoint (e.g., 84.4 points (±16.2) for social exclusion and 72.3 points (±22.6) for treatment impact) and therefore provides an appropriate reference frame for paediatric chronic illness samples [28]. Comparable mid-to-upper range DCGM-37 values have also been reported in other paediatric chronic disease contexts, including Chinese paediatric oncology [29], children and adolescents with physical disability or chronic disease participating in sport [42], and Scandinavian validation cohorts [43], collectively demonstrating that DCGM-37 values in paediatric chronic conditions frequently cluster above simple scale midpoints.

For pain-attitude measures, it is informative to consider related paediatric instruments from the same theoretical family, such as the paediatric Survey of Pain Attitudes. The original development studies in adolescents with physical disability [44,45] demonstrated mid-range clustering of subscale means and satisfactory internal consistency. These paediatric Peds-SOPA reports therefore provide a relevant comparative framework for interpretation of our PAQ-27 results. Direct paediatric normative values for the specific PAQ-27 subscales used in our study (stoicism and cautiousness) are not available. For context, however, community adult samples reported mean stoicism scores of 3.13 (±0.62) and cautiousness 2.68 (±0.60) [29], whereas chronic pain adult cohorts reported stoicism 3.34 (±0.59) and cautiousness 2.94 (±0.66) [46]. This range therefore provides a reasonable external comparative anchor.

In our sample, both DCGM-37 and PAQ-27 subscale means lie approximately at the midpoint of their respective possible ranges. Whilst direct cross-study comparison is necessarily limited by differences in age, diagnosis, and cultural context, the above DISABKIDS and paediatric pain-attitude literature indicates that our findings are broadly consistent with prior paediatric chronic illness and chronic pain cohorts; DCGM-37 values are not indicative of extreme impairment, and PAQ-27 results fall in the mid-range of published pain-belief scores.

The DCGM-37 subscales for independence, physical limitations, emotion, and social inclusion clearly favoured the PG. Both groups started at comparable baseline levels; however, by the end of the study, the PG demonstrated substantially better outcomes, reporting greater independence, fewer physical limitations, fewer negative emotions related to disability, and higher levels of social inclusion. Importantly, the magnitude of these effects was not only statistically significant, but also clinically meaningful. Within-group effect sizes (Cohen’s d, Table 4) in the PG were consistently large across these domains (d = 1.41 for independence; 1.86 for physical limitations; 1.78 for emotion; 1.80 for social inclusion). In contrast, the no-PG showed only small-to-moderate effect sizes in the same domains (d = 0.31, 0.85, 0.68, and 0.44, respectively), indicating that even where *p*-values reached significance, the magnitude of change was considerably smaller. This is fully consistent with the observed absolute change values. For no-PG, mean change scores were 2.10, −4.78, −4.10, and 2.58 points—reflecting statistical significance without obvious clinical relevance. In contrast, the PG demonstrated substantially larger changes—10.41, −9.81, −11.99, and 8.72 points—representing improvements two to five times greater, and supported by large effect sizes. Thus, the combination of change scores, effect sizes, and *p*-values converges to a consistent interpretation: the PG experienced clinically meaningful improvement, whereas the no-PG did not.

No statistically or clinically important differences were observed for the social exclusion and treatment impact subscales of the DCGM-37, where effect sizes were very small in both groups.

Turning to pain attitudes, as assessed by the PAQ-27, we observed a broadly consistent pattern with that seen in the responsive DCGM-37 subscales. It is worth noting here that he stoicism subscale reflects a tendency to downplay pain, suppress its expression, and maintain a belief in one’s superior ability to control it, while the cautiousness subscale captures uncertainty in judging whether an unpleasant sensation constitutes pain. Both groups began at comparable baseline levels; however, by the end of the intervention, the PG achieved significantly higher values across both PAQ dimensions. Within-group effect sizes reinforce the clinical relevance of these findings. The PG showed very large effect sizes for both stoicism (d = 2.38) and cautiousness (d = 1.84), whereas the no-PG demonstrated smaller effect sizes (d = 0.46 and 0.22, respectively). Thus, although statistical significance was also observed for the no-PG, the magnitude of change was minimal and unlikely to reflect meaningful clinical benefit. This is consistent with the absolute change values: in no-PG, mean increases were only 0.20 points for stoicism and 0.13 points for cautiousness, while the PG showed substantially larger shifts of 0.80 and 0.83 points, respectively, representing four- to seven-fold greater improvement. Hence, conclusions for PAQ are fully aligned with those observed for DCGM-37.

Taken together, the consistent pattern across both quality-of-life outcomes and pain attitudes suggests that transient injuries and their associated pain were not merely tolerated but may have initiated positive psychosocial change. Adolescents in the PG develop—or began to develop—a more resilient and self-reliant approach to discomfort, while also learning to calibrate their appraisal of bodily signals. This aligns with contemporary resilience theory, which posits that repeated exposure to tolerable, bounded stressors fosters adaptive coping and the strengthening of regulatory processes rather than vulnerability [47,48,49]. In other words, sport-related pain, when experienced within a supportive and supervised environment, appears to foster adaptive forms of toughness without tipping into maladaptive denial [50,51,52]. From a biopsychosocial perspective, this pattern is consistent with models of pain in which appraisal, affective interpretation, behavioural response, and meaning-making are central modulators of pain-related disability, particularly in youth [53,54,55]. Notably, the benefits observed in the DCGM-37 were concentrated in subscales assessing domains of quality of life—namely, independence, perception of physical limitations, emotions linked to disability, and sense of social inclusion—that reside ‘within the individual’ and are largely independent of external influences. This suggests that sport-related pain may scaffold internal model-building processes characteristic of adolescent development [56,57], where identity, autonomy, and emotion regulation systems are undergoing rapid maturation. This pattern suggests that responses to sport-related pain may first emerge in the internal world of the individual, initiating the development of a resilient or challenge-oriented mindset referenced in the Introduction. Importantly, our findings provide the first evidence that such transformation can be fostered in young people whose life attitudes and emotional frameworks are still forming. Exposure to manageable adversity during this sensitive developmental window may have lasting consequences, shaping resilience and commitment that extend beyond sport. This observation directly addresses the knowledge gap highlighted in the Introduction, a topic that has received limited attention in the existing literature.

No differences were observed for the social exclusion and treatment impact DCGM-37 subscales, and several explanations may account for this finding. First, sport participation, injuries, and associated pain may primarily exert effects within the individual, without substantially influencing aspects of quality of life that are shaped by the external environment. Regarding social exclusion, it is notable that most training sessions occurred during school holidays, whereas children with disabilities are most at risk of experiencing social exclusion within the school setting. Consequently, these effects may have been attenuated in our study, and conducting the research during the academic year might have revealed different outcomes. With respect to treatment impact, children with disabilities are often exposed to frequent therapeutic interventions and may perceive therapy negatively, regardless of its specific purpose. In our study, the therapy provided was aimed at alleviating sport-related pain rather than addressing the underlying disability, yet participants may have regarded it as ‘just another therapy’, diminishing its perceived benefit.

Although inherently positive, the changes observed in the PAQ-27, which favoured the PG, may carry potential risks. Increased stoicism, as captured by the PAQ-27, or a ‘can-do’ mindset can sometimes lead to elevated risk-taking and neglect of injury-prevention behaviours, particularly in the absence of adequate monitoring and education. Instruments assessing the relationship between risk, pain, and injury (e.g., the Risk–Pain–Injury Questionnaire) and empirical studies have identified associations between permissive pain attitudes and unsafe escalation of training load or premature return-to-play decisions [58]. To mitigate such hazards, structured injury-education and safe-practice programmes for athletes, coaches, and support staff are essential. Concussion education programmes and targeted return-to-sport interventions provide a precedent for effective knowledge translation in youth sport [59,60]. In our programme, we have introduced brief modules on symptom recognition, graded training, and help-seeking. These represent practical translational steps that can curb maladaptive risk-taking while preserving the psychosocial benefits observed in participants.

Finally, the practical relevance of the observed improvements in health-related quality of life and pain attitudes is reflected in follow-up data. In the subsequent season, adolescents from the PG were twice as likely to return to sitwake training as those from the no-PG (65% vs. 29%). Although this difference is noteworthy, causality cannot be inferred. Factors such as higher baseline motivation, stronger engagement, or greater perceived benefits of participation may also have contributed. Thus, while experiencing and overcoming pain might have strengthened commitment to sport in some individuals, this interpretation remains tentative and should be explored in future studies.

### Limitations

Several limitations of this study should be acknowledged. First, our findings pertain to small- to moderate-intensity pain, and it remains unclear how participants would respond to more severe injuries. Indeed, among study dropouts, two participants withdrew even after minor injuries, suggesting that higher-intensity injuries might result in greater attrition.

Second, the study was limited to sitwake, which constrains generalisability to other sports. Nonetheless, it is reasonable to assume that sport-related pain and minor injury may evoke broadly similar internal responses across disciplines, as the critical factor is the experience of injury within the performance context rather than the specific sport. Limitations regarding generalisability also apply to age and type of disability: the sample predominantly comprised young participants with mild impairments, and responses may differ in older athletes or those with more severe disabilities.

Third, this study employed a case–control design. Although prospective in nature, it provides only Level 3 evidence, as classified by the Oxford Centre for Evidence-Based Medicine Levels of Evidence.

Finally, the sample size was relatively small. However, given the three-year duration of the study and the niche nature of the sport, this represents a substantial achievement. Furthermore, the sample met the pre-specified criteria for statistical power, supporting the reliability of the results obtained. However, we acknowledge that a relatively small sample size, especially for subgroup analyses, may have limited the statistical power of some comparisons; therefore, replication in larger cohorts is warranted to strengthen the generalizability of our findings.

## 5. Conclusions

Participation in sitwake training accompanied by transient injuries and moderate pain may yield positive psychosocial outcomes in adolescents with lower-limb disabilities. Participants who experienced injuries (PG) demonstrated greater improvements in health-related quality of life, as measured by the DCGM-37—particularly independence, physical limitations, emotions, and social inclusion—together with more adaptive pain attitudes, reflecting increased resilience and self-reliance. Improvements observed in the no-PG were smaller and of limited clinical relevance. No significant changes were detected for social exclusion or treatment impact, which may be attributable to the internalised nature of these constructs and contextual factors such as school holidays and prior therapeutic exposure.

A higher proportion of PG participants returned to sitwake training in the subsequent season (65% vs. 29%). Although it is likely that manageable pain may have contributed to continued participation, other factors, e.g., individual baseline motivation and enjoyment of training, could also have played a role.

Overall, these findings suggest that structured exposure to manageable sport-related pain can foster resilience, adaptive coping, and continued engagement in sport in adolescents with lower-limb disabilities.

## Figures and Tables

**Table 1:** Demographic characteristics in the two research groups.

	Pain Group	No-Pain Group	
	Mean ± SD (Min–Max) or Number (%)	Mean ± SD (Min–Max) or Number (%)	*p* Pain vs. No-Pain
number	20	34	-
age (years)	14.45 ± 2.06 (12.00–18.00)	14.00 ± 1.86 (12.00–18.00)	0.31 !
body height (m)	1.58 ± 0.11 (1.41–1.81)	1.54 ± 0.10 (1.42–1.80)	0.15 !
body mass (kg)	51.40 ± 11.28(36.00–76.00)	47.90 ± 9.71 (35.00–74.00)	0.24 !
body mass index (kg/m^2^)	20.32 ± 1.58 (17.85–23.62)	20.09 ± 1.39 (17.12–22.84)	0.57 !
	male: 12 (60.00)	male: 20 (58.80)	
sex	female: 8 (40.00)	female: 14 (41.20)	0.91 †

! Student’s *t*-test; † Chi^2^ test.

**Table 2:** Type and anatomical location of injuries in the pain group. The total number of injuries exceeds the number of participants. Two individuals (10%) sustained injuries at different sites on separate occasions, while five participants (25%) experienced multiple injuries simultaneously (three participants (15%) at two sites and two participants (10%) at three sites). Percentages in the table refer to the total number of injuries.

		Number (%)
	wrist flexor strain/tendonitis	3 (10.3)
	wrist extensor strain/tendonitis	1 (3.4)
	biceps strain/tendonitis	2 (6.9)
Type:	rotator cuff strain	7 (24.2)
soft tissue injuries	lower back strain	2 (6.9)
	hand joint sprains	2 (6.9)
	wrist sprain	4 (13.8)
	shoulder capsule stress	6 (20.8)
Type:	neck sprain	1 (3.4)
joint injuries	sacroiliac joint sprain	1 (3.4)
	hand, wrist, and forearm	10 (34.6)
	arm and shoulder	15 (51.7)
	neck and upper torso	1 (3.4)
injury location	lower torso	3 (10.3)
multiple injuries	-	9 (31.0)

**Table 3:** The standardised 13-week sitwake training programme—stages, goals, and content.

Stage	Goal	Content
I. Adaptation and safety on the short cable (2.0)	Safety Basic position Body control	Week 1: familiarisation with equipment, water, and water start technique; Week 2: starts from the platform, short rides at low speed; Week 3: maintaining a straight line, changing the rope grip
II. Control and turns on the long cable (5.0)	Smooth riding in a straight line Control when changing rope direction	Week 4: mastering turn technique around pylons; Week 5: longer rides, rhythm (intervals: faster-slower); Week 6: smooth direction changes, edge-changing technique on a straight line
III. Improving technique, first tricks	Balance training Preparation for tricks	Week 7: playful elements (one-handed riding, quick grip changes); Week 8: learning slide riding, first spins; Week 9: freestyle elements (riding on and off a small obstacle)
IV. Sport elements and competition	Developing skills in competition conditions	Weeks 10–11: improving tricks—turns, small jumps, freestyle adapted to ability; Week 12: competition preparation
V. Summary and testing	Progress assessment Group integration, Motivation for further training	Week 13: competition simulation (contest runs), video analysis, individual assessment setting goals for the future

**Table 4:** Subscale scores and change scores for the DISABKIDS Chronic Generic Module (DCGM-37) and Pain Attitudes Questionnaire (PAQ-27) in the pain and no-pain groups.

		Pain Group	No-Pain Group
	1	56.69 ± 6.97 (45.83–70.83)	57.88 ± 7.19 (45.83–75.00)
DCGM-37	2	67.10 ± 7.74 (54.17–79.17)	59.98 ± 6.43 (45.83–75.00)
independence	Δ	10.41 ± 3.44 (4.16–16.66)	2.10 ± 3.12 (−4.17–8.34)
	1	49.38 ± 4.74 (41.67–58.33)	49.27 ± 5.75 (37.50–58.33)
DCGM-37	2	39.57 ± 5.80 (33.33–50.00)	44.48 ± 5.50 (33.33–54.17)
physical limitations	Δ	−9.81 ± 3.10 (−16.67–−4.17)	−4.78 ± 2.74 (−8.34–0.00)
	1	54.13 ± 5.88 (43.43–64.28)	55.56 ± 5.64 (42.86–64.28)
DCGM-37	2	42.14 ± 7.56 (32.14–57.14)	51.47 ± 6.47 (35.71–64.28)
emotion	Δ	−11.99 ± 4.37 (−17.86–−3.57)	−4.10 ± 4.32 (−10.72–7.14)
	1	52.10 ± 6.99 (33.33–62.67)	49.16 ± 7.88 (25.00–62.67)
DCGM-37	2	50.83 ± 6.84 (33.33–58.33)	49.53 ± 7.04 (33.33–62.67)
social exclusion	Δ	−1.27 ± 3.86 (−8.33–4.17)	0.37 ± 4.29 (−4.34–8.50)
	1	57.54 ± 4.85 (50.00–66.67)	57.38 ± 5.65 (45.83–66.67)
DCGM-37	2	66.27 ± 4.84 (54.17–70.83)	59.97 ± 6.09 (45.83–70.83)
social inclusion	Δ	8.72 ± 4.86 (0.00–16.67)	2.58 ± 3.56 (−4.17–8.50)
	1	60.04 ± 6.83 (45.83–70.83)	58.99 ± 5.47 (50.00–66.67)
DCGM-37	2	58.15 ± 8.18 (45.83–75.00)	56.89 ± 8.22 (41.67–70.83)
treatment impact	Δ	−1.89 ± 4.94 (−8.50–4.17)	−2.10 ± 7.39 (−12.67–12.50)
	1	2.55 ± 0.41 (1.86–3.50)	2.53 ± 0.45 (1.79–3.43)
PAQ-27	2	3.36 ± 0.27 (2.79–3.86)	2.73 ± 0.42 (2.14–3.50)
stoicism	Δ	0.81 ± 0.27 (0.21–1.21)	0.20 ± 0.23 (−0.21–0.64)
	1	2.58 ± 0.51 (1.62–3.46)	2.78 ± 0.55 (1.92–3.92)
PAQ-27	2	3.40 ± 0.40 (2.69–3.92)	2.90 ± 0.63 (1.69–4.46)
cautiousness	Δ	0.83 ± 0.32 (0.31–1.54)	0.13 ± 0.26 (−0.38–0.69)

1/2—baseline/follow-up measurement (mean ± standard deviation [min–max] (in points)); Δ—change score: follow-up minus baseline score.

**Table 5:** Statistical significance and effect sizes for the intra- and inter-group differences in subscale scores, as well as change scores for the DISABKIDS Chronic Generic Module (DCGM-37) and Pain Attitudes Questionnaire (PAQ-27).

		Pain Group	No-Pain Group	
		*p* 1 vs. 2 (d)	*p* 1 vs. 2 (d)	*p* Pain vs. No-Pain (d)
	1			0.555 ! (0.17)
DCGM-37	2	<0.001 !* (1.41)	<0.001 !* (0.31)	0.001 !* (1.00)
independence	Δ	…	…	<0.001 †* (2.53)
	1			0.120 † (0.02)
DCGM-37	2	<0.001 * (1.86)	<0.001 ^* (0.85)	0.003 †* (0.87)
physical limitations	Δ	…	…	<0.001 †* (1.72)
	1			0.380 ! (0.25)
DCGM-37	2	<0.001 !* (1.78)	<0.001 ^* (0.68)	<0.001 †* (1.33)
emotion	Δ	…	…	<0.001 †* (1.81)
	1			0.228 † (0.39)
DCGM-37	2	0.164 ^ (0.18)	0.657 ^ (0.05)	0.488 † (0.19)
social exclusion	Δ	…	…	0.229 † (0.40)
	1			0.941 † (0.03)
DCGM-37	2	<0.001 ^* (1.80)	0.001 ^* (0.44)	<0.001 †* (0.97)
social inclusion	Δ	…	…	<0.001 †* (1.46)
	1			0.516 † (0.17)
DCGM-37	2	0.103 ! (0.25)	0.095 ^ (0.31)	0.611 † (0.15)
treatment impact	Δ	…	…	0.986 † (0.03)
	1			0.706 † (0.05)
PAQ-27	2	<0.001 !* (2.38)	<0.001 ^* (0.46)	<0.001 †* (1.83)
stoicism	Δ	…	…	<0.001 !* (2.44)
	1			0.741 ! (0.38)
PAQ-27	2	<0.001 !* (1.84)	0.007 !* (0.22)	0.003 !* (0.97)
cautiousness	Δ	…	…	<0.001 !* (2.41)

* statistically significant; ! Student’s *t*-test; † Mann–Whitney test; ^ Wilcoxon test; 1/2—baseline/follow-up measurement; Δ—change score; d—Cohen’s d coefficient.

## Data Availability

The raw data supporting the conclusions of this article will be made available by the authors on request.

## Abbreviations

The following abbreviations are used in this manuscript:

DCGM-37	37-item DISABKIDS Chronic Generic Module Questionnaire
PAQ-27	27-item Pain Attitudes Questionnaire
PG	Pain Group
no-PG	no-Pain Group
